# Isolation and characterization of novel microsatellite markers and their application for diversity assessment in cultivated groundnut (*Arachis hypogaea*)

**DOI:** 10.1186/1471-2229-8-55

**Published:** 2008-05-15

**Authors:** Luu M Cuc, Emma S Mace, Jonathan H Crouch, Vu D Quang, Tran D Long, Rajeev K Varshney

**Affiliations:** 1 International Crop Research Institute for the Semi Arid Tropics (ICRISAT), Patancheru- 502 324, Greater Hyderabad, Andhra Pradesh, India; 2 Agriculture Genetics Institute, Vietnamese Academy Agriculture Science, Van Dien, Thanh Tri, Hanoi, Vietnam; 3 Department of Primary Industries & Fisheries, Hermitage Research Station, 604 Yangan Road, Warwick, QLD 4370, Australia; 4 International Wheat and Maize Improvement Centre (CIMMYT), Apdo. Postal 6-641, 06600 Mexico, D.F., Mexico; 5 Vietnamese Academy Agriculture Science, Van Dien, Thanh Tri, Ha Noi, Vietnam

## Abstract

**Background:**

Cultivated peanut or groundnut (*Arachis hypogaea *L.) is the fourth most important oilseed crop in the world, grown mainly in tropical, subtropical and warm temperate climates. Due to its origin through a single and recent polyploidization event, followed by successive selection during breeding efforts, cultivated groundnut has a limited genetic background. In such species, microsatellite or simple sequence repeat (SSR) markers are very informative and useful for breeding applications. The low level of polymorphism in cultivated germplasm, however, warrants a need of larger number of polymorphic microsatellite markers for cultivated groundnut.

**Results:**

A microsatellite-enriched library was constructed from the genotype TMV2. Sequencing of 720 putative SSR-positive clones from a total of 3,072 provided 490 SSRs. 71.2% of these SSRs were perfect type, 13.1% were imperfect and 15.7% were compound. Among these SSRs, the GT/CA repeat motifs were the most common (37.6%) followed by GA/CT repeat motifs (25.9%). The primer pairs could be designed for a total of 170 SSRs and were optimized initially on two genotypes. 104 (61.2%) primer pairs yielded scorable amplicon and 46 (44.2%) primers showed polymorphism among 32 cultivated groundnut genotypes. The polymorphic SSR markers detected 2 to 5 alleles with an average of 2.44 per locus. The polymorphic information content (PIC) value for these markers varied from 0.12 to 0.75 with an average of 0.46. Based on 112 alleles obtained by 46 markers, a phenogram was constructed to understand the relationships among the 32 genotypes. Majority of the genotypes representing subspecies *hypogaea *were grouped together in one cluster, while the genotypes belonging to subspecies *fastigiata *were grouped mainly under two clusters.

**Conclusion:**

Newly developed set of 104 markers extends the repertoire of SSR markers for cultivated groundnut. These markers showed a good level of PIC value in cultivated germplasm and therefore would be very useful for germplasm analysis, linkage mapping, diversity studies and phylogenetic relationships in cultivated groundnut as well as related *Arachis *species.

## Background

The cultivated peanut or groundnut, *Arachis hypogaea *L., (2n = 4*x *= 40) is a major crop in most tropical and subtropical areas of the world, with 68% of groundnut cultivated world-wide produced in Asia (23 Mt), 24% in Africa (8 Mt) and the remaining 8% (3.5 Mt) from North America, the Caribbean, Europe and Oceania [1]. The seeds are used for direct human consumption, and as an oil and protein source [2]. Additionally, plant residues are extremely important as fodder for cattle in many regions of the world [3]. The crop is becoming increasingly important as an income source in tree plantations before tree crops mature. In Africa and Asia, groundnut is intercropped between maize, sorghum, and soybean or, in a few areas, between mature coconut trees [4].

In contrast to the wealth of phenotypic diversity observed within cultivated groundnut, the genetic diversity observed to date within the cultivated gene-pool is much lower. This low level of genetic variation in cultivated groundnut is attributed to its origin from a single polyploidization event that occurred relatively recently on an evolutionary time scale [5]. However, additional contributing factors to the low levels of molecular polymorphism observed to date could be the marker techniques used and the amount of diversity of samples tested [6].

Molecular markers, in general, and microsatellites or simple sequence repeats (SSRs) in particular have proven very useful for crop improvement in many species [7]. In groundnut, the use of molecular markers for breeding applications, however, has been limited by the low level of the genetic variation in this species. Nevertheless, in recent years, significant efforts have been made to develop the SSR markers in groundnut [8-10]. Development of SSR markers traditionally requires cloning and sequencing and hence is more cost and labour-intensive, compared to PCR arbitrary priming techniques e.g. randomly amplified polymorphic DNAs (RAPDs), amplified fragment length polymorphism (AFLP) [7]. However, once the SSR markers are developed, their applications in breeding activities particularly using high throughput approaches becomes very cost effective. To isolate the SSRs from genomic DNA libraries, several approaches for creating SSR-enriched genomic libraries have been developed, with SSR selection either before [11-13] or after genomic library construction [14].

By using different approaches, > 500 SSRs have been developed in groundnut [15]. By using these SSR markers, good progress has been made in developing the genetic maps and diversity studies in AA- and BB-genome groundnut species [8,9,16-22]. In case of cultivated germplasm, however, these SSR markers showed very low level of polymorphism [8,19-22]. This is one of the reasons that despite the availability of moderate number of SSR markers in groundnut, not a single genetic map based on cultivated germplasm has been published so far. To overcome the low level of polymorphism, one of simple solutions will be to develop a critical number of SSR markers in groundnut so that a repertoire of about 200–300 polymorphic SSR markers for cultivated groundnut germplasm may be available.

The present study was initiated in order to isolate and characterize new microsatellite markers from groundnut, following a microsatellite enriched genomic library approach. The overall aim of this study is to enhance the repertoire of polymorphic SSR markers for cultivated groundnut germplasm so that genetic mapping and trait mapping could be feasible in cultivated groundnut.

## Results and Discussion

### SSR-enriched library

The SSR enriched library was constructed from the genotype TMV2 following by modified method of Fischer and Bachmann [23]. This library was enriched for CA and CT SSR repeat motifs. From this library, 3,072 clones were picked from 32 96-well plates. Hybridization of these clones with digoxigenin-labeled SSR probes (CA and CT) provided 720 (23.4%) putatively positive clones. Sequencing of these clones indicated the insert size in the range of 50 bp to 792 bp with an average size of 309 bp. Majority of clones (43.9%) contained the insert of moderate size (200 bp-400 bp) while 34.6% clones contained small inserts (50 bp-200 bp) and 21.5% clones contained inserts of > 500 bp.

Analysis of sequence data mentioned above with Tandem Repeat Finder (TRF) had 490 (68%) clones which contained one or more SSRs. The efficiency of the enrichment procedure for the constructed library was higher as compared to other SSR isolation studies of groundnut. Like the present study, 61% of clones were found to contain SSRs in the study of He and colleagues [20], 56% clones had SSRs in the study of Gimenes and colleagues [18] and 43.7% clones were reported to contain SSRs by Wang and colleagues [24]. However very low enrichment efficiency (10% to 31%) were obtained in some other libraries enriched for SSRs [8,9,21]. Indeed, this enrichment efficiency depends on many factors including the choice of restriction enzyme used for library construction, the SSR probes used for enrichment, etc. The approach used in the present study seems to be the most efficient enrichment strategy for SSR isolation in groundnut.

A redundancy level of 26% in the SSR-enriched genomic library was observed through multiple sequence alignment analysis using the *ClustalW *programme; in total 5 copies of one clone was observed, 4 copies of five clones, 3 copies of 10 clones and 2 copies of 65 clones. The rate of redundant SSR-containing clones was found to be comparable (26%) to other studies utilizing microsatellite-enriched genomic libraries in other plant species, e.g., olive tree (*Olea europaea *L., 16.6%) [25], onion (*Allium cepa *L. 24.3%) [23]. As compared to SSR isolation studies in groundnut where upto 67% redundancy has been observed [8,20,21], the strategy employed in the present study seems to be quite effective to isolate a higher proportion of novel and unique SSRs. Observed level of redundancy in this study could be explained due to the existence of multiple copies of some SSRs in the groundnut genome, which may be present on both the A and B genomes within cultivated *A. hypogaea*. The bias observed for some SSRs being repeated in multiple clones could also be explained by the fact that during the enrichment procedure (adaptor ligation, amplification of single-strand enriched DNA, bacterial growth before plating) some fragments can be arbitrarily selected over the rest.

Despite the addition of excess adapter during the enrichment procedures, 7.2% of clones were identified as concatenates, generated during the initial restriction/ligation step, by the presence of internal *Rsa*I and *Mlu*I restriction sites. Another type of concatenation may be formed during the PCR step of the enrichment cloning procedure [27]. Such chimeras usually remain undetected and may result in the failure of some primer pairs to amplify genomic DNA templates in the evaluation of primers [28].

### Occurrence and features of SSRs

Sequence analysis of 720 clones showed the presence of one or more SSRs in 490 (68%) clones. Following the definitions of Weber [29], 71.2% of the SSRs identified were perfect, 13.1% were imperfect (when SSRs are interrupted by few bases) and 15.7% were compound (when more than one SSRs are spaced by few base pairs) with 9.8% being compound perfect and 5.9% being compound imperfect. Similar kind of distribution of different SSR classes was observed in different SSR isolation studies in groundnut [8,9,18].

In terms of the repeat motifs, the GT/CA repeat motif was the most common, accounting for 37.6% of all repeat types, followed by GA/CT repeat at 25.9%. The previous surveys carried out on microsatellite abundance in plant genomes have shown AT as the most frequently occurring dinucleotide repeat motifs followed by AG/CT and GT/CA [30-34]. The AT repeat is self-complementary and is difficult to screen for by colony hybridization, hence the library was not enriched for AT. Abundance of GA/CT, GT/CA, AT and ATT repeat motifs in isolated SSRs in groundnut in the present study is in agreement with earlier reports on isolation of SSRs in groundnut [8,9,20,24]. The use of separate GA and GT filters could increase the ability of detecting perfect GA/CT and GT/CA repeat motifs or the frequency of the repeats, in comparison to using mixtures of different repeat motifs in the same hybridization. However several studies have shown the retrieval of higher proportion of compound SSRs (upto 75%) when the library was enriched using a mixture of different SSR oligos [18,35,36].

The maximum repeat unit number of dinucleotide repeat motifs of GT/CA and GA/CT were 48 and 50 units, respectively; the overall repeat motif number ranging from 7 to 50. In fact, in some studies, the markers developed for longer repeat motifs were found more informative for detection of polymorphism in cultivated groundnut germplasm [9]. In addition to GT and GA repeats containing SSRs, several SSRs containing the repeat motifs- (AAG)_n_, (CAA)_n_, (TAA)_n_, (TTG)_n_, (GTT)_n_, (TTC)_n_, (CCT)_n_, (AAAG)_n_, (TTTC)_n_, (TTCTC)_n_, (CTTTT)_n_, (CTCTTT)_n _and (GTGTTT)_n _with 2–11 repeat numbers were also isolated. It was interesting to note that most of the clones containing these repeats had an additional repeat of GT/CA or GA/CT. Gimenes and colleagues [18] also observed 37% SSRs with different repeats, like in the present study, that were not totally complementary sequences to the oligonucelotide probes used. However, the repeat motif ATT is highly abundant and informative in several legume species like soybean [37], chickpea [38] and pea [39] was not observed abundant in the present study. In case of groundnut, many reports are available on isolation and distribution of SSRs, only two studies [8,9] indicated the abundance of AAT repeat motifs. In the present study, as only 720 clones sequenced were selected randomly from the set of 3072 clones, probably sequencing of larger number of clones could have showed the abundance of AAT repeat motifs.

### Marker development

SSR containing sequences were used for primer designing using Primer3 programme. Following the standard criteria: primer length- 18–27 bp; Tm- 57–63°C; GC content – 40–60%, maximum Tm difference between forward and reverse primer – 1.5°C, primer pairs were designed for 170 SSR containing clones [see Additional file 1]. Of this set, 47.1% primer pairs were designed for perfect repeats, 18.2% for imperfect repeats while the remaining 34.7% for compound repeats. For the remaining sequences, the primer designing could not be possible as in some cases sequence quality was poor while in some cases the SSRs were too near the start or end of the insert. The percentage of primers designed, in relation to the number of clones sequenced (23.6%) is higher than some studies in groundnut like Moretzsohn and colleagues [9] (10.5%), He and colleagues [20] (14.0%) and Ferguson and colleagues [8] (21.3%) while lower than some other reports such as Moretzsohn and colleagues [21] (41.4%) and Wang and colleagues [24] (43.7%). This may be attributed to the size range of insert, the restriction enzyme used for genomic DNA library construction and the approach used for SSR enrichment, etc. [7].

Newly designed SSR markers were tested for amplification on two genotypes i.e. TMV2 and ICG 99001. Of this set, only 104 (61.2%) primer pairs yielded the scorable amplicon in the genotypes examined (Table 1). The functionality of the primer pairs is comparatively lower than the studies of Ferguson and colleagues [8] and Moretzsohn and colleagues [9] who observed amplification in 84.9% and 81.6% cases, respectively. It is noteworthy that several PCR profiles and PCR optimization strategies were adopted in above mentioned studies, however in the present study to save costs and time, PCR conditions for non-amplifiable markers were not optimized repeatedly. Out of 104 working primers, 89 (85.6%) primer pairs were optimized on 65°C -60°C touch down profile, 14 (13.5%) primer pairs optimized on 60°C -55°C touch down profile while only one (0.9%) primer pair was optimized on 55°C -45°C specific profiles. It is quite likely to increase the rate of functionality of newly developed markers by using different PCR conditions and profiles.

**Table 1:** Overview on SSR marker development and polymorphism

**Repeat unit classes**	**Markers designed**	**Marker yielding amplification**	**Markers showing polymorphism**
5 – 10	15	11 (73.3%)	3 (27.3%)
11 – 15	37	27 (72.9%)	14 (51.8%)
16 – 20	56	38 (73.2%)	23 (56.1%)
21 – 25	33	16 (48.5%)	4 (25.0%)
26 – 30	11	6 (54.5%)	1 (16.7%)
31 – 35	7	3 (42.9%)	1 (33.3%)
36 – 40	5	2 (40.0%)	0
> 40	6	1 (16.7%)	0

Total	170	104 (61.2%)	46 (44.2%)

It is also noted that the markers for less than 20 repeat units produced amplicons in about 73% cases, while the markers containing the higher number of repeat units (> 20) yielded amplicon in 16.7% – 54.5% cases only. It is possible that higher number of repeat units make the *Taq *polymerase unstable that makes it unable to extend alongwith the template DNA [7].

### SSR polymorphism

In order to assess the potential of newly developed SSR marker for detecting the polymorphism in 4× groundnut genotypes, all the 104 primer pairs yielding PCR products were tested on the set of 32 genotypes (Table 2). As a result, only 46 (44.2%) markers showed polymorphism in the germplasm analyzed (Table 3). Of the 46 primers, 30 primers were for perfect SSRs while 16 primers were for imperfect SSRs. Marker polymorphism observed in the present study is higher or comparable to earlier studies on SSR diversity in cultivated groundnut germplasm. For example, in two different studies, He and colleagues observed polymorphism with 29.2% [40] and 33.9% markers [20] while 35.8% markers showed polymorphism in the study of Moretzsohn and colleagues [9]. In all these studies, the SSR markers, like in the present study, were isolated from the genomic DNA libraries. It seems that either the SSR markers developed in the present study are more informative or the germplasm surveyed here is more diverse. Higher informativeness of the newly developed markers is supported by the general theory that degree of polymorphism of the SSR marker increases with the total length of the repeat [7,9,29]. The majority of the markers (> 80%) developed here contained more than 10 repeat units for the corresponding SSRs (Table 1). However, Ferguson and colleagues [8] observed higher (48.7%) marker polymorphism as compared to this study which can be attributed to the diverse nature of the germplasm examined in their study.

**Table 2:** Details on germplasm used for diversity analysis

**S.No.**	**Genotype**	**Botanical Variety**	**Country of origin**	**Market type**
1	ICG 4389	*hypogaea*	India	virginia, runner or bunch
2	ICG 10362	*hypogaea*	Nigeria	virginia, runner or bunch
3	ICG 10971	*hypogaea*	Peru	virginia, runner or bunch
4	ICG 12235	*hypogaea*	Bolivia	virginia, runner or bunch
5	ICG 12621	*hypogaea*	India	virginia, runner or bunch
6	ICG 13420	*hypogaea*	Chad	virginia, runner or bunch
7	ICGV 99003	*hypogaea*	India	virginia bunch
8	ICGV 99005	*hypogaea*	India	virginia bunch
9	ICG 15405	*hirsuta*	Peru	peruvian runner
10	ICG 15419	*hirsuta*	Peru	peruvian runner
11	ICG 3204	*fastigiata*	China	valencia
12	ICG 9987	*fastigiata*	Bolivia	valencia
13	ICG 10704	*fastigiata*	China	valencia
14	ICG 11605	*fastigiata*	Bolivia	valencia
15	ICG 13430	*fastigiata*	Chad	valencia
16	ICG 14421	*fastigiata*	Nigeria	valencia
17	ICGV 99004	*fastigiata*	India	valencia
18	ICG 6284	*fastigiata*	USSR	-
19	ICG 405	*fastigiata*	Paraguay	-
20	ICG 10074	*peruviana*	Peru	-
21	ICG 10911	*peruviana*	Peru	-
22	ICG 1705	*peruviana*	Peru	-
23	ICG 7898	*aequatoriana*	Ecuador	-
24	ICG 12553	*aequatoriana*	Ecuador	-
25	ICG10384	*vulgaris*	Nigeria	spanish
26	ICG 11175	*vulgaris*	Bolivia	spanish
27	ICG 11505	*vulgaris*	China	spanish
28	ICG 11515	*vulgaris*	China	spanish
29	ICG 12483	*vulgaris*	Peru	spanish
30	ICG 13415	*vulgaris*	Chad	spanish
31	ICGV 99001	*vulgaris*	India	spanish
32	TMV 2	*vulgaris*	India	spanish

In recent years, SSR markers have been developed from the expressed sequence tags (ESTs) because of increasing the emphasis on developing the functional molecular markers [10]. Luo and colleagues [22] had 20% of the markers showing polymorphisms; while Moretzsohn and colleagues [9] detected polymorphisms in cultivated germplasm with 7.5% of the markers. Lower level of polymorphism of EST-based SSR markers can be attributed to their origin from highly conserved proportion of the genome [41]. In our opinion, the crops like groundnut having narrow genetic background needs higher number of SSR markers derived from genomic DNA library instead from cDNA library or ESTs.

The numbers of alleles detected by the set of 46 polymorphic markers were in the range of 2 to 5 with an average of 2.44 alleles per locus (Table 3). The PIC value for these polymorphic markers varied from 0.12 (IPAHM 92) – 0.75 (IPAHM 123) with an average of 0.46 (Table 3). When looking at SSR classes and motifs, the trinucleotide SSRs showed higher allele numbers (average 2.5 per locus) and PIC values (average 0.53 per marker) followed by dinucleotide (average alleles- 2.45 per locus; PIC value- average 0.45 per marker) and compound SSRs (average alleles- 2.35 per locus; PIC value- average 0.44 per marker). Among dinucleotide SSRs, GA/CT repeat motifs exhibited more informativeness (average alleles- 2.6 per locus and PIC value- average 0.50 per marker) as compared to GT/CA repeat motifs (average alleles- 2.0 per locus and PIC value- average 0.33 per marker). Ferguson and colleagues [8] as well as Moretzsohn and colleagues [9] observed higher informativeness of GA/CT repeat motifs. Therefore to develop more polymorphic markers for cultivated groundnut, we propose to isolate and develop the GA/CT repeat based SSR markers.

**Table 3:** Polymorphism features of SSR markers developed

**S.No**	**Marker**	**No. of alleles**	**PIC**
1	IPAHM 23	2	0.40
2	IPAHM 73	3	0.62
3	IPAHM 82	2	0.34
4	IPAHM 92	2	0.12
5	IPAHM 93	3	0.60
6	IPAHM103	5	0.73
7	IPAHM 105	3	0.62
8	IPAHM 108	3	0.62
9	IPAHM 123	4	0.75
10	IPAHM 136	2	0.49
11	IPAHM 147	2	0.27
12	IPAHM 165	3	0.51
13	IPAHM 166	2	0.48
14	IPAHM 171 a	3	0.55
15	IPAHM 171 c	2	0.17
16	IPAHM 176	2	0.49
17	IPAHM 177	2	0.31
18	IPAHM 219	2	0.36
19	IPAHM 229	2	0.46
20	IPAHM 282	2	0.43
21	IPAHM 283	2	0.49
22	IPAHM 287	3	0.63
23	IPAHM 288	2	0.43
24	IPAHM 290	2	0.26
25	IPAHM 352	3	0.58
26	IPAHM 354	3	0.57
27	IPAHM 356	3	0.51
28	IPAHM 373	3	0.58
29	IPAHM 375	2	0.63
30	IPAHM 395	2	0.48
31	IPAHM 406	2	0.36
32	IPAHM 407 a	2	0.30
33	IPAHM 429	2	0.35
34	IPAHM 451	2	0.13
35	IPAHM 455	2	0.31
36	IPAHM 468	2	0.32
37	IPAHM 475	3	0.67
38	IPAHM 524	2	0.48
39	IPAHM 531	2	0.48
40	IPAHM 540	2	0.25
41	IPAHM 569	2	0.38
42	IPAHM 659	4	0.69
43	IPAHM 684	2	0.46
44	IPAHM 689	2	0.50
45	IPAHM 716	3	0.70
46	IPAHM 718	2	0.12

**Figure 1:**
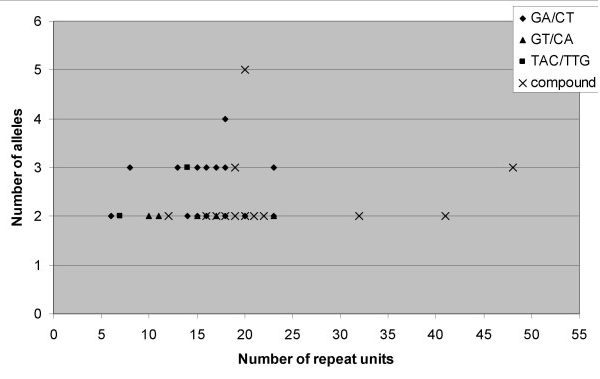
Number of alleles per locus for SSR markers of different repeat units.

**Figure 2:**
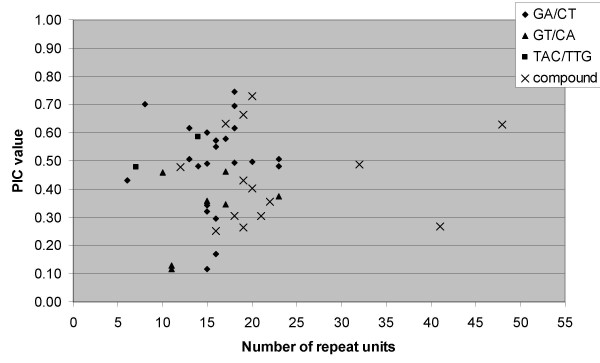
Relationships between PIC value of SSR markers and repeat unit length.

To understand the possible relationship between polymorphism of SSR markers with the repeat unit length of the corresponding SSRs, two scatter plots were made between repeat unit length and number of alleles detected (Fig. 1) and the PIC value calculated (Fig. 2). The scatter plot between number of alleles and repeat unit length shows the widest variation in number of alleles was between 13 and 20 repeats and a lower number of alleles found in the low number or very high number repeats. However it does not provide any conclusive relationships between the number of alleles and repeat unit length as indicated by Ferguson and colleagues [8] and Moretzsohn and colleagues [9] that loci with longer repeats are much more likely to be more variable. Indeed, among the polymorphic SSR markers, the IPAHM 147 marker containing highest number of repeat units (41) provided just 2 alleles while the IPAHM 103 markers with 20 repeat units long SSR revealed the highest number of alleles (5). This is possible as the majority of the polymorphic SSR markers detected 2 and 3 alleles in the present study.

The scatter plot between PIC value and repeat unit length indicates that the higher PIC values (> 0.50) were between 13 and 20 repeats while lower PIC values were found in the low number (< 13) or very high number (> 20) repeats. It is noteworthy that the relationship appeared to be SSR class specific as it was more consistent for the compound SSRs. As no other report, to the best of our knowledge, is available on relationship between PIC value and repeat unit length in groundnut, a direct comparison of the observed results could not be possible.

Based on our investigations on relationship of repeat unit length with number of alleles or PIC value, no consistent relationship between the number of repeat units and SSR polymorphism was observed. It has been reported earlier that the degree of polymorphism increases with the total length of the repeat [9,29,38,39], some other studies including in groundnut showed no relationship or weak correlation between SSR polymorphism and repeat unit length [8,20,42,43].

### Diversity analysis and genetic relationships

**Figure 3:**
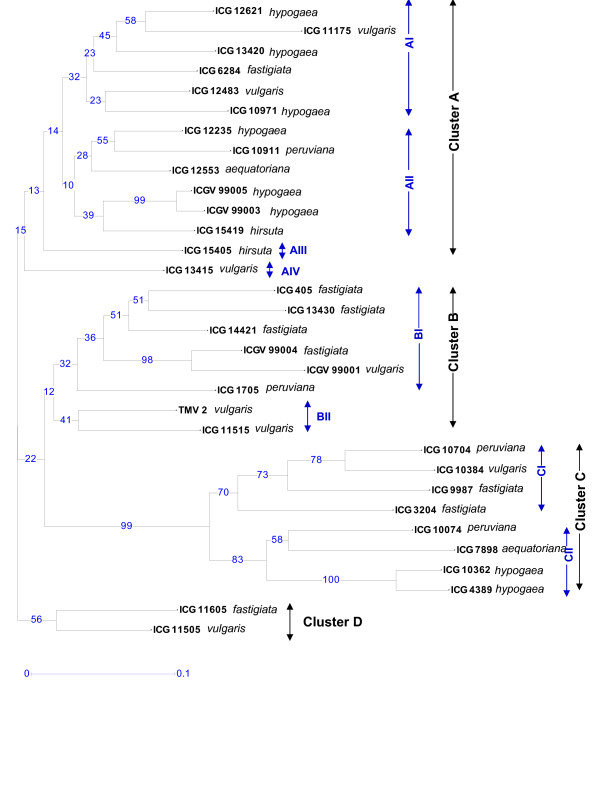
**Dendrogram based on allele sharing genetic distances of 32 cultivated groundnut genotypes generated by the neighbor joining analysis method.** The numbers on the branches indicate bootstrap values (expressed in percentages, based on 100 replications).

Based on the unique DNA fingerprint profiles of each accession of cultivated groundnut obtained by the polymorphic markers, a phenogram was constructed to understand the relationships among the cultivated groundnut germplasm surveyed. The dendrogram based on DICE similarity coefficient and constructed using the DARwin programme classified the germplasm in four main clusters A, B, C and D (Fig. 3). The cluster A contained 14 genotypes, the cluster B contained 8 genotypes while the other two clusters namely C and D contained 8 and 2 genotypes, respectively. Under each of these main clusters, genotypes were grouped further into sub clusters. For instance, the cluster A contained four subclusters (AI, AII, AIII and AIV) and the cluster B (BI, BII) and C (CI and CII) contained two subclusters each.

Majority of the genotypes (8 out of 10) representing subspecies *hypogaea *(6 *hypogaea *and 2 *hirsuta *genotypes) were found in the main cluster A. The genotypes belonging to *fastigiata *subspecies were grouped mainly under the main clusters B and C. The grouping of genotypes representing two subspecies in different clusters agrees with the classification of groundnut botanical varieties [44]. However, in an earlier study [45] genotypes representing the two subspecies were classified in only two groups.

The cluster B contained 4 (out of 10) genotypes belonging to variety *fastigiata *and three genotypes belonging to variety *vulgaris*. The cluster C also contained the genotypes from the *fastigiata *subspecies that includes 2 genotypes of variety *fastigiata*, 2 to variety *peruviana *and one each from varieties *vulgaris *and *aequatoriana*. The cluster D contained only 2 genotypes belonging to subspecies *fastigiata*. Majority of the nodes under the main clusters were supported by high bootstrap values.

It is important to note that positioning of botanical varieties *aequatoriana *and *peruviana *to the subspecies *fastigiata *or *hypogaea *has been debatable in the literature. For instance, in the past, based on morphological and physiological traits, two botanical varieties were classified under the subspecies *fastigiata *(that includes other varieties *fastigiata *and *vulgaris*) while AFLP markers suggested relationships of *aequatoriana *and *peruviana *to *hypogaea *rather than subspecies *fastigiata *[45]. The present study included only two *aequatoriana *and three *peruviana *genotypes, and they were grouped under all three major clusters (A, B and C). Nevertheless, like the observations of He and Prakash [45], four out of the five genotypes representing *aequatoriana *and *peruviana *subspecies showed closer relationships with the *hypogaea *genotypes (clusters AII and CII) while only one genotype of *peruviana *subspecies (ICG 1705) showed some proximity to subspecies *fastigiata*. The botanical variety *peruviana*, based on RAPD and ISSR marker data, was classified as an operational taxonomic unit in addition to four varieties i.e. *fastigiata, vulgaris *(both belonging to subspecies *fastigiata*), *runner *and *bunch *(both belonging to subspecies *hypogaea*) [46].

It is interesting to note that the two accessions with resistance to leaf rust (ICGV 99003 and ICGV 99005) were grouped together (AII) and similarly the other two accessions resistance to late leaf spot (ICGV 99001 and ICGV 99004) were grouped together (BI). It seems that genotypes resistant to leaf rust and late leaf spot shared the pedigree or have the same resource of resistance. However, three accessions resistance to early leaf spot (ICG 405, ICG 1705 and ICG 6284) were scattered in the dendrogram. The accession, TMV2, susceptible to all three diseases (leaf rust, late leaf spot and early leaf spot) was present under cluster B. The dendrogram suggests the potential parental genotypes having higher genetic diversity for constructing the mapping population(s) for mapping the leaf rust, late leaf spot and early leaf spot. Even selection and utilization of diverse cultivars in breeding programmes is needed to enhance the diversity of breeding populations for selection gains in the future [47].

## Conclusion

The results of this study highlight a reliable and efficient way of obtaining microsatellites markers from cultivated groundnut. It is desirable to isolate and characterize more DNA markers in cultivated groundnut for more productive genomic studies, such as genetic mapping, marker-assisted selection, and gene discovery. Construction and sequencing of SSR enriched library yielded a total of 400 SSRs, however, primer pairs could be designed for only 170 SSRs of which 104 primer pairs showed the functionality. As a result, the present study contributes a new set of 104 SSR markers for cultivated groundnut. In order to assess the potential of newly developed markers for germplasm analysis, screening of these markers on 32 genotypes showed reasonable level of polymorphism. Newly developed markers detected on average 2.44 alleles per locus with an average PIC value 0.46. The present study also provided some indications about nature and type of repeat class or length of SSRs on the polymorphism of corresponding SSR marker. Finally, the SSR markers, developed in this study would be very useful for germplasm analysis, population genetic structure and phylogenetic relationships.

## Methods

### Plant material

For constructing the SSR-enriched genomic libraries, the groundnut germplasm line TMV2, belonging to the Spanish botanical variety was used. While two genotypes (TMV2 and ICG 99001) were used for optimizing the PCR assays for newly developed SSR markers, a set of 32 genotypes were used for identifying the potential polymorphic markers for cultivated groundnut germplasm (Table 2). Of these 32 genotypes, 10 genotypes represent the subspecies *hypogaea (*2 to variety *hirsuta *and 8 to variety *hypogaea) *and the remaining 22 genotypes belong to subspecies *fastigiata *(10 to variety *fastigiata*, 7 to variety *vulgaris*, 3 to variety *peruviana *and 2 to variety *aequatoriana*).

#### DNA extraction

Total genomic DNA was isolated from unfurled leaves according to a modified CTAB-based procedure [48]. The quality of DNA was checked on 1% agarose gels and the DNA concentrations using spectrophotometer UV- 160A following the recommendations of manufacturer (Shimadzu Corporation, Japan).

#### Construction of SSR-enriched library

A modified protocol of Fischer and Bachmann [23] was used for constructing the SSR enriched library. Six micrograms of genomic DNA genomic were digested by blunt end – generating restriction endonuclease *Rsa*I. After confirming digestion on agarose gel electrophoresis, the *Mlu*I adapter, consisting of a 21-mer (5'-CTCTTGCTTA**CGCG**TGGACTA-3') and a phosphorylated 25-mer (5'-pTAGTCCA**CGCG**TAAGCAAGAGCACA-3') was ligated to the blunt termini of restriction fragments using 50 ng adapter/μg of genomic DNA. Ligation was performed for 2 hours at 37°C in order to allow the restriction digest to continue, hence preventing the DNA fragments from re-ligating to one another. The ligation products were then separated on a 1% TAE agarose gel and fragments size from 100–900 bp and 900–1500 bp cut from the gel and purified with GFX Gel Band Purification Kit (Amersham Biosciences, USA). The constructs were then heat denatured and hyribridised to biotinylated microsatellite oligonucleotides. The hybridizations were carried out using 75 μl of 6×SSC and 150 nM of each biotinylated oligo (GT)_15 _and (GA)_15 _overnight at T_hyb_= T_m_-5°C. The hybrids were subsequently bound to streptavidin – coated magnetic beads (Dynabeads M -280 Streptavidin- Dynal, Norway). In order to capture the target sequences, the beads were incubated either at room temperature for 15 min for the 100–900 bp fragments or at 43°C for one hour for the 900–1500 bp fragments. Non-hybridizing genomic DNA was subsequently removed through a series of washes; twice in 2× SSC; 0.1% SDS (5 mins each, at 25°C), twice in 1× SSC (5 mins each, at 25°C) and finally twice in 1×SSC at T_hyb _for 2 and 5 mins respectively. The bound DNA was eluted as single stranded fragments in TE preheated to 95°C.

The hybridized DNA fragments served as a template for PCR using the 21-mer oligonucleotide as the primer (30 cycles with 56°C annealing temperature). Following PCR, like samples were combined and purified using the GFX column purification Kit (Amersham Biosciences, USA). The purified PCR products were then digested with *Mlu*I to obtain vector-compatible, sticky ended fragments by incubation at 37°C overnight. The restriction fragments were purified using a MicroSpin™ column (Amersham Biosciences, USA) prior to ligation into a modified pUC19 vector (pJV1) (Edwards et al., 1996 [11]) which had been linearized with *Bss*HII and dephosphorylated. The ligated vector fragments were transformed into competent *E. coli *DH5α cells (Invitrogen, USA), plated on LB agar containing ampicillin (100 μg/ml). To allow for blue-white selection, the plates were spread with 5-bromo-4-chloro-3-indolyl-β-D-galactopyranoside (X-gal; 80 μg/ml) plus isopropyl β-D-thiogalactopyranoside (IPTG; 80 μg/ml). White colonies were picked and plated in a grid on LB containing ampicillin, prior to making colony lifts with Nylon Membranes, positively charged, following the recommendation of manufacturer (Amersham Biosciences, USA). Hybridization was carried out at 42°C overnight using digoxigenin-labelled probes containing the SSR motifs being searched (Roche, Germany).

#### Sequencing of SSR-positive clones

The SSR positive clones identified after hybridization were grown overnight in 3 ml LB broth with 100 μg/mL ampicillin. Plasmid DNA was extracted using GFX™ Micro Plasmid Prep Kit (Amersham Biosciences, USA). Subsequently, the plasmid DNA was sequenced using M13 Forward 24-mer Sequencing Primer following the dideoxynucleotide chain termination method on ABI 3700 sequencer. Base calling was carried out using Phred [49]. Sequence data were quality trimmed using the sliding windows of 50 bp with a minimal average Phred score of 20.

#### SSR identification and primer designing

The sequencing data were analysed using the *ClustalW *programme in order to determine the rate of redundancy in the library. Non-redundant sequences were analysed with Tandem Repeat Finder software [50]. The SSR containing sequences were subsequently used for primer design using Primer3 programme. Primers were designed from within the regions flanking the repeat motifs; for dinucleotides the repeat motifs selected were greater than 14 bp in length, trinucleotides greater than 15 bp and tetranucleotides greater than 16 bp.

### Amplification and visualization of microsatellite loci

PCR reactions for all the primer pairs were performed in 5 μl reaction volume following three touch down profiles i.e. 65–55°C (89 markers), 60–55°C (14 markers) and 55–45°C (1 marker). The PCR was performed on 5 ng of genomic DNA with varying amount of primer pairs, Mg^2+^, dNTPs and *Taq *DNA polymerase. Details on these reaction components for each primer pair (marker), that yielded PCR amplicon, are given in Additional file 1. Touch down amplification programs included 94°C for 2 min, 30 cycles of 94°C for 45 sec, annealing temperature (65–55°C/60–55°/55–45°C) for 60 sec, 72°C for 60 sec and a final extension of 10 min at 72°C.

The PCR products were separated on a non- denaturing 6% polyacrylamide gel at 250 V for 2.5 to 3 hours in 1× TBE buffer and visualized by silver staining, modified from Kolodny [51]. The presence or absence of amplicons in the genotypes examined was scored as 1 or 0, respectively.

#### Statistical analysis

The polymorphism information content (PIC) of each microsatellite locus was determined as described by Weir [52]:

PIC = 1-Σ *P*_i_^2^,

where *P*_i _is the frequency of the *i*th allele in the genotypes examined.

Allelic data obtained in 0–1 fashion for all alleles at microsatellite loci amplified were used for computing the inter-individual genetic dissimilarity following simple matching coefficient by using DARwin v 5.0.153 programme [53]. The dissimilarity matrix thus generated was further used to generate UPGMA (Unweighted Pair Group Method with Arithmetic mean) dendrogram following neighbour-joining (NJ) by using the DARwin programme.

## Authors' contributions

LMC and ESM executed majority of the research work, VDQ, TDL and RKV analyzed the data. JHC, ESM and RKV were involved in designing and planning the work and interpreting the results. RKV drafted and edited the manuscript critically with help of LMC. All authors read and approved the final manuscript.

## Supplementary Material

Additional file 1 Features and polymorphism status of new set of SSR markers developed. The data provided represent the details of the new set of SSR markers e.g. marker name, Genbank accession IDs, primer sequences, PCR conditions and amplification status. Click here for file
