# Supplementary material for: Isolation and characterization of novel microsatellite markers and their application for diversity assessment in cultivated groundnut (Arachis hypogaea)
Source: BMC Plant Biol. 2008 May 15;8:55. doi: 10.1186/1471-2229-8-55 (PMC2416452; doi:10.1186/1471-2229-8-55)
Supplement: Additional file 1 — Features and polymorphism status of new set of SSR markers developed. The data provided represent the details of the new set of SSR markers e.g. marker name, Genbank accession IDs, primer sequences, PCR conditions and amplification status. [file 1471-2229-8-55-S1.doc]

**Additional file 1: Features and polymorphism status of new set of SSR markers developed**

| **Marker ID** | **Genbank accession ID** | **Repeat motif** | **Primer sequence (5’ - 3’)**  **Forward (F) and reverse (R)** | **PCR profile; *Reaction component** | **Expected size (bp)** | **Amplification status** |
| --- | --- | --- | --- | --- | --- | --- |
| IPAHM 23 | ER974415 | (CA)17(TA)3 | F: GTGTCTTTTCGTTCGCGATT | 60-55; 1 | 130 | Polymorphic |
|  |  |  | R: CGACTCTTAGGGTGGATTATAGTAAGA |  |  |  |
| IPAHM 33 | ER974416 | (GT)19 | F: CATCTTGTTGGTCGCTGCTA |  | - | No amplification |
|  |  |  | R: TGTGGAATTGTGAGCGGATA |  | - |  |
| IPAHM 40 | ER974417 | (AC)14TTCACC (CA)32 | F: CCAGCTTGCATGGCTCTG |  | - | No amplification |
|  |  |  | R: GCTTCTTCTTCAACGCTCCTAGT |  |  |  |
| IPAHM 41 | ER974418 | (TC)14 | F: CTTAATACGCCGCCTCACC | 60-55; 1 | 400 | Monomorphic |
|  |  |  | R: TCCTTTTTGGTTTTGGAAGC |  |  |  |
| IPAHM 44 | ER974419 | (CA)48 | F: GGCCAAACAAATCAGGAGAG |  | - | No amplification |
|  |  |  | R: GGGCACAAAATTAAGGGGATA |  |  |  |
| IPAHM 56 | ER974420 | (GT)40 | F: TGGATGTTTATGGAGAGAGAGAGA |  | - | No amplification |
|  |  |  | R: ATGACCATGAGTACGCCAAG |  |  |  |
| IPAHM 58 | ER974421 | (GT)7GC(GT)5GC (GT)11 | F: GATGGACTACCCTCGCCTTT |  | - | No amplification |
|  |  |  | R: TGTGGAATTGTGAGCGGATA |  |  |  |
| IPAHM 67 | ER974422 | (TA)4(TG)24 | F: TGGAACATTATTCCCATTGC |  | - | No amplification |
|  |  |  | R: CACCCCAGGCTTTACACTTT |  |  |  |
| IPAHM 73 | ER974423 | (GA)13 | F: ACTAACAGCGTAGCCGTCGT | 65-60; 1 | 170 | Polymorphic |
|  |  |  | R: ACCCCTCTCAGTGTTTTCCA |  |  |  |
| IPAHM 75 | ER974424 | C6(TC)18 | F: ATATGGCATGGGTGTTCATC | 65-60; 4 | 200 | Monomorphic |
|  |  |  | R: CCTAAAGCAGCATATTGCATAC |  |  |  |
| IPAHM 79 | ER974425 | (GT)22 | F: TCATTGATTAGAGAGTGTTGTGAGG | 65-60; 2 | 480 | Monomorphic |
|  |  |  | R: TGTGGAATTGTGAGCGGATA |  |  |  |
| IPAHM 82 | ER974426 | (GA)15 | F: CCATATCATAGCCGCCAAGT | 65-60; 4 | 350 | Polymorphic |
|  |  |  | R: TACATCCACGATGCAGAAGG |  |  |  |
| IPAHM 88 | ER974427 | (GT)3(TG)19 | F: GTGTTGCAGTGGCAAAGATG | 65-60; 4 | 225 | Monomorphic |
|  |  |  | R: TCAGCCTGCGAAACTAAGGT |  |  |  |
| IPAHM 89 | ER974428 | (TG)20 | F: TCTCGGTTAAATGCCCCTAGTA |  | - | No amplification |
|  |  |  | R: GAGCGGATAACAATTTCACACA |  |  |  |
| IPAHM 91 | ER974429 | (GA)15GGAAGA | F: GGAGAACTCACCGGAATTGA |  | - | No amplification |
|  |  |  | R: CAAAGCCGTCATTACAACTACAC |  |  |  |
| IPAHM 92 | ER974430 | (GT)11 | F: CTTCATGTCTGATTAGCAGAAGGT | 65-60; 2 | 200 | Polymorphic |
|  |  |  | R: CCCTGATATGGCCTCTTCAA |  |  |  |
| IPAHM 93 | ER974431 | (CT)15 | F: TCCATCGTTAGTGGCACTGT | 65-60; 4 | 200 | Polymorphic |
|  |  |  | R: GTCGACTCCTGCCCAATCTA |  |  |  |
| IPAHM 94 | ER974432 | (GA)50 | F: GCTTTCGCTGGGAGAATACA | 60-55; 4 | 550 | Monomorphic |
|  |  |  | R: GCGGTGACATCCATCTCTCT |  |  |  |
| IPAHM 96 | ER974433 | (GA)10AA(GA)6GG (GA)23 | F: ACCGTCTTTCACCTCACCAC | 65-60; 5 | 400 | Monomorphic |
|  |  |  | R: CAAAGCCGTCATTACAACTACAC |  |  |  |
| IPAHM 97 | ER974434 | (AC)11(TC)18 | F: TCAGCTGTCTCTCTGCTCCA | 65-60; 3 | 400 | Monomorphic |
|  |  |  | R: CTCCAAGCAAAAAGTTGAAGG |  |  |  |
| IPAHM 98 | ER974435 | (CT)20 | F: TTCCGTGTGACAGAAATACCC | 60-55; 1 | 200 | Monomorphic |
|  |  |  | R: GAAGAGTGGTGGGTGGAAGA |  |  |  |
| IPAHM 122 | ER974445 | (CT)8CC(CT)5C (CT)6 | F: TCTCTCCCACACAAACCACA |  | - | No amplification |
|  |  |  | R: CTGGGAGGAAGAGAGAGCTG |  |  |  |
| IPAHM 100 | ER974436 | (GA)22 | F: GGCTTGGAGAAAAGAGAGCA | 65-60; 5 | 110 | Monomorphic |
|  |  |  | R: CAAAGAACAGGGACACATACCA |  |  |  |
| IPAHM 103 | ER974437 | (CA)3(GA)17 | F: GCATTCACCACCATAGTCCA | 65-60; 2 | 160 | Polymorphic |
|  |  |  | R: TCCTCTGACTTTCCTCCATCA |  |  |  |
| IPAHM 105 | ER974438 | (CT)18 | F: CAGAGTTTGGGAATTGATGCT | 65-60; 5 | 300 | Polymorphic |
|  |  |  | R: GCCAGATCTGAGCAAGAACC |  |  |  |
| IPAHM 108 | ER974439 | (TC)18 | F: CTTGTCAAACTCTGTGACTTAGCA | 60-55 | 230 | Polymorphic |
|  |  |  | R: CATGAACAATTACACCCAGTCA |  |  |  |
| IPAHM 109 | ER974440 | (GA)9 | F: TGCGAAACTGAAACAACCAG |  | - | No amplification |
|  |  |  | R: GTAGTGGTGCGTGGGTTTCT |  |  |  |
| IPAHM 111 | ER974441 | (TG)9CG(TG)15 | F: TGAACCAGAGATATCTGAAAAGTTG | 65-60; 3 | 300 | Monomorphic |
|  |  |  | R: ACCCGTTAGCAACAACCAAA |  |  |  |
| IPAHM 113 | ER974442 | (TC)21 | F: CTACGACATTGGCGGTGAC | 65-60; 3 | 210 | Monomorphic |
|  |  |  | R: CTGTGAATGGTGAAGGAAGAAG |  |  |  |
| IPAHM 117 | ER974443 | (CT)14 | F: CTCAAGAAAACCGCCATCTC | 65-60; 1 | 195 | Monomorphic |
|  |  |  | R: TCTCCAACACCATTCTTCCA |  |  |  |
| IPAHM 121 | ER974444 | (CT)19(CA)15 | F: GCTATATCCAGACAGCCATCG | 65-60; 3 | 180 | Monomorphic |
|  |  |  | R: GAGAAGGGTGGATTGAAGAAGA |  |  |  |
| IPAHM 123 | ER974446 | (GA)18 | F: CGGAGACAGAACACAAACCA | 65-60; 1 | 190 | Polymorphic |
|  |  |  | R: TACCCTGAGCCTCTCTCTCG |  |  |  |
| IPAHM 124 | ER974447 | (CT)16 | F: AACCACCTAAGACGCGATTC | 65-60; 3 | 420 | Monomorphic |
|  |  |  | R: GCGGCAATAGTAGTGGCAAT |  |  |  |
| IPAHM 125 | ER974448 | (AG)14 | F: GAAATAGGGATGAAGAAATGTGAGA |  | - | No amplification |
|  |  |  | R: TATTCCAAACCCTAGGATCTTTACG |  |  |  |
| IPAHM 129 | ER974449 | (GA)9CAAA(GA)21 | F: CGGTGATTGGATAGAATAAGGA | 65-60; 1 | 110 | Monomorphic |
|  |  |  | R: GGTCTTCTTCCCACATGTCC |  |  |  |
| IPAHM 130 | ER974450 | (GA)13 | F: GAAGGAGACAGATGGAATTGG | 65-60; 5 | 180 | Monomorphic |
|  |  |  | R: AGCTTCTGCGTTCTTCCAAC |  |  |  |
| IPAHM 132 | ER974451 | (GA)19GGGA(GGA)2 | F: GTTTCAGAGCCTGGGGAATC | 65-60; 4 | 400 | Monomorphic |
|  |  |  | R: CCCTCTCTCCACCACTACCA |  |  |  |
| IPAHM 136 | ER974452 | (TC)2(CT)13 | F: CCCCTTTCTCCACTACTACCA | 65-60; 5 | 300 | Polymorphic |
|  |  |  | R: TTCTCCTAGGGACTCCGATG |  |  |  |
| IPAHM 137 | ER974453 | (GA)15 | F: GAGAGCCTGGGGATCCTG |  | - | No amplification |
|  |  |  | R: ATGCGGCATGCACTCTTAAC |  |  |  |
| IPAHM 139 | ER974454 | (GA)30GG(GA)5 | F: GAATTGAGAGAGAAAGAACCGAGT |  | - | No amplification |
|  |  |  | R: CTTCCATGGTCCTCTCCTCA |  |  |  |
| IPAHM 142 | ER974455 | (TG)13(AG)10 | F: CCCACATTAATCCTAACCAAGA |  | - | No amplification |
|  |  |  | R: TCGCAACTCAAGTTAGTCCA |  |  |  |
| IPAHM 146 | ER974456 | (GA)18G4 | F: CTGGGAGGAAGAGAGAGCTG | 60-55; 4 | 750 | Monomorphic |
|  |  |  | R: CACGCGTGGACTAACTTGTAA |  |  |  |
| IPAHM 147 | ER974457 | (TC)14TT(TC)4 | F: CCATTGTCTTCCTCCTCTATCTC | 65-60; 3 | 120 | Polymorphic |
|  |  |  | R: TGTTGATGCAGCAATTAGGC |  |  |  |
| IPAHM 150 | ER974458 | (GA)8AA(GA)3AA (GAA)3 | F: GGGAGGGTGGTGTGAGAGTA |  | - | No amplification |
|  |  |  | R: TAACGTTTGGGTGGTTCTCC |  |  |  |
| IPAHM 161 | ER974459 | (GA)16 | F: TGCCAATAGAGAGAACACTACCA |  | - | No amplification |
|  |  |  | R: AATGCCTTAAGCCGATGTTG |  |  |  |
| IPAHM 162 | ER974460 | (TC)10 | F: ACTACCCTTCCCTCCACCAC |  | - | No amplification |
|  |  |  | R: ATGAGAGTCTGGGGATCCTG |  |  |  |
| IPAHM 164 | ER974461 | (GA)20 | F: TTGAAAGGGGGTGAATGAAG | 65-60; 1 | 175 | Monomorphic |
|  |  |  | R: TGCTTTGTGCTTAATGGAAGG |  |  |  |
| IPAHM 165 | ER974462 | (GA)13 | F: CAACACGTTCGCTTCCAGAT | 65-60; 1 | 220 | Polymorphic |
|  |  |  | R: TCACTCTCATTTCCGCCATT |  |  |  |
| IPAHM 166 | ER974463 | (CAA)9(TAA)3 | F: GGACAATTATGCCCCTCAGC | 65-60; 1 | 180 | Polymorphic |
|  |  |  | R: TCCTTCCTCTGAGCTTTTCG |  |  |  |
| IPAHM 167 | ER974464 | (GA)17 | F: CACTCCCCATCAAAACTAACAG | 60-55; 1 | 120 | Monomorphic |
|  |  |  | R: TTCCTCCTTCCCCAAGAGAT |  |  |  |
| IPAHM 168 | ER974465 | (GAAA)4(GA)12CA (GA)4 | F: AGAAGCGGAAAGAAATGAAGG |  | - | No amplification |
|  |  |  | R: GCCGTCATTACAACTACACTACCA |  |  |  |
| IPAHM 171a | ER974466 | (TC)7TGTT(TC)9 | F: TTGGTTGTTCGTAGCTCTGC | 65-60; 5 | 200 | Polymorphic |
|  |  |  | R: AGCACGGCAAACACTAACACT |  |  |  |
| IPAHM 171b | ER974467 | (CT)3(CTTT)2(CT)14 | F: CTAAATTCAAACCGCGAACC |  | - | No amplification |
|  |  |  | R: TGAGAAGATCCACAATGCTGA |  |  |  |
| IPAHM 171c | ER974468 | (GA)16 | F: CAACACAAGCCCACAACAAA | 65-60; 2 | 145 | Polymorphic |
|  |  |  | R: TCCATCATCACCCTCATCAA |  |  |  |
| IPAHM 176 | ER974469 | (GA)18 | F: TTCAGCAAAAACATGCAAGG | 65-60; 3 | 200 | Polymorphic |
|  |  |  | R: TGCAATGAGTTATATTCACCTCTCC |  |  |  |
| IPAHM 177 | ER974470 | (CA)11TA(CA)3 (TA)4 | F: TCAGCGGAGAAGAAAACTAAGG | 65-60; 2 | 190 | Polymorphic |
|  |  |  | R: GAGGTGTTTGGAGAACTAGGATTT |  |  |  |
| IPAHM 178 | ER974471 | (GA)7CA(GA)16 | F: TTCTTCTTTGCTCGAAGATCC |  | - | No amplification |
|  |  |  | R: CATGGAAGAAATGTTGCTCA |  |  |  |
| IPAHM 207 | ER974472 | (TG)20 | F: AGCGCCTCATCACCTTTAAT |  | - | No amplification |
|  |  |  | R: CCTAACTCAGCCTGCGAAAC |  |  |  |
| IPAHM 213 | ER974473 | (CA)19 | F: GCGAAACTAAGGTTGGTCGT |  | - | No amplification |
|  |  |  | R: GGGATGGAGAAAGGGAAAAG |  |  |  |
| IPAHM 215 | ET190211 | (CA)23 | F: CCTAACTCAGCCTGCGAAAC |  | - | No amplification |
|  |  |  | R: TGCATGTGTGTGTGTGTGTG |  |  |  |
| IPAHM 219 | ER974474 | (TG)15 | F: TCTCTTTTGTGTATTTTGGGCTA | 65-60; 5 | 130 | Polymorphic |
|  |  |  | R: AGCCTGCGAAACTAAGGTTG |  |  |  |
| IPAHM 229 | ER974475 | (CA)14TA(CA)3 | F: TCAGCCTGCGAAACTAAGGT | 65-60; 1 | 140 | Polymorphic |
|  |  |  | R: TGGAGAACTAGGATCTCTTTTGTG |  |  |  |
| IPAHM 245 | ER974476 | (GT)13 | F: CCCAAGGACCTAGTGACCAA | 65-60; 1 | 290 | Monomorphic |
|  |  |  | R: GGACCCTTAGCACATTCCAA |  |  |  |
| IPAHM 254 | ER974477 | (GA)5AA(GA)20CT (GA)4 | F: TGAAATGGTGGCTTGCAATA |  | - | No amplification |
|  |  |  | R: TGCTGAGTCGCTCATTCTCT |  |  |  |
| IPAHM 255 | ER974478 | (AGGG)3(AG)23 | F: AGGAGGGAGACGAGAGAAGG |  | - | No amplification |
|  |  |  | R: GGGGTTGTCACTGCCATAAT |  |  |  |
| IPAHM 263 | ER974479 | (GA)14GG(GA)38 | F: GAGAGCCTGGGGATCCTG |  | - | No amplification |
|  |  |  | R: TCTCCCTCTCTCTCTCTCTGTCTG |  |  |  |
| IPAHM 267 | ER974480 | (CT)17 | F: CACTTCTCTGCTGCCCAAAT | 60-55; 4 | 130 | Monomorphic |
|  |  |  | R: CAGCCCCTTACACCACTCAT |  |  |  |
| IPAHM 269 | ER974481 | (TG)14 | F: TGTTGATTTCTGTTGCACACT |  | - | No amplification |
|  |  |  | R: CTCGTATGTTGTGTGGGAGT |  |  |  |
| IPAHM 270 | ER974482 | (TG)8CG(TG)15 | F: AACCAGGTATCTGAAAAGTTGTCC |  | - | No amplification |
|  |  |  | R: ACCCGTTAGCAACAACCAAA |  |  |  |
| IPAHM 271a | ER974483 | (TCC)3(TTC)3 | F: TTGGGTGGTTCTCCAAAACT | 65-60; 2 | 195 | Monomorphic |
|  |  |  | R: TGCAGGGAGGAAAGAAAAAG |  |  |  |
| IPAHM 271b | ER974484 | (GT)12(GA)15 | F: CGTGCTTACTTCATATGCTTTG |  | - | No amplification |
|  |  |  | R: TCGCTGCCCTGTGTACTAA |  |  |  |
| IPAHM 272 | ER974485 | (TA)7N(GT)12TA (TG)7 | F: TCCACTTTGGAGAAACAGGTG | 60-65 | 300 | Monomorphic |
|  |  |  | R: CAAACCCCTGACTCGACCT |  |  |  |
| IPAHM 273 | ER974486 | (TC)8TT(TC)7 | F: GATCGTCGCTTCTTTGCTCT | 65-60; 5 | 120 | Monomorphic |
|  |  |  | R: CCTTCCTAGAACAGCGATGG |  |  |  |
| IPAHM 281 | ER974487 | (GA)17 | F: GACTCAGCAGCCCTAAAACG | 60-55; 5 | 250 | Monomorphic |
|  |  |  | R: TCAGTGTGCCTTACAAAGAATCA |  |  |  |
| IPAHM 282 | ER974488 | (CA)14(TA)5 | F: AAGCCTTTGCGAATATAACCA | 65-60; 3 | 180 | Polymorphic |
|  |  |  | R: TGCAGGACTTGTATTTTGAGGA |  |  |  |
| IPAHM 283 | ER974489 | (TA)4(TG)26TTG (GT)2 | F: GAAGACAAACCCCTCTGCTG | 65-60; 5 | 120 | Polymorphic |
|  |  |  | R: TCGGATAGCATGGATGTGAA |  |  |  |
| IPAHM 284 | ER974490 | (CT)3CC(CT)18(CA)15 | F: GCTTTTGCCACAAACAAACA |  | - | No amplification |
|  |  |  | R: ACCCACATTAATCCTAACCAAGA |  |  |  |
| IPAHM 285 | ER974491 | (AG)14G(AG)8 | F: GGGCCCAAGATCCAACATA |  | - | No amplification |
|  |  |  | R: GGGAACTGAAATCGCATTGT |  |  |  |
| IPAHM 287 | ER974492 | (TG)16(AG)22 | F: TCTAACCCTTCGGTTCATGG | 65-60; 5 | 200 | Polymorphic |
|  |  |  | R: TCACTATCCCATCCCTGCTC |  |  |  |
| IPAHM 288 | ER974493 | (GA)4AA(GA)2 | F: AAGTGAATTTGGGGATGCTG | 65-60; 5 | 190 | Polymorphic |
|  |  |  | R: CTCCACCACTGCCACTATCA |  |  |  |
| IPAHM 290 | ER974494 | (TA)3(CA)3CC(CA)5 (TA)8 | F: CCACCGCTGATGTGTAATTGTA | 65-60; 1 | 300 | Polymorphic |
|  |  |  | R: GACGTGTAGTTGAAAACAACAGTATCA |  | - |  |
| IPAHM 29 | ER974495 | TTTC(TTCTC)4 | F: CACTTCCACTTGCATCTCCA | 65-60; 5 | 250 | Monomorphic |
|  |  |  | R: CCATGGCTGCTTTCTAGGAG |  |  |  |
| IPAHM 295 | ER974496 | (GA)20 | F: TGCTGGAATGGTAACTTTGCT | 60-55; 2 | 140 | Monomorphic |
|  |  |  | R: TAACCCCCTTCACTCCCTCT |  |  |  |
| IPAHM 297 | ER974497 | (TC)18 | F: AATGCCAAGCTAGATAGAGTAGTGA | 65-60; 3 | 210 | Monomorphic |
|  |  |  | R: CAGCGAATAGAGCAGAAGGA |  |  |  |
| IPAHM 302 | ER974498 | (AG)14G(AG)8 | F: ACCACACCCCTCACTCACTC | 65-60; 5 | 150 | Monomorphic |
|  |  |  | R: ACTCCCACGTTGTTGCTGTC |  |  |  |
| IPAHM 307 | ER974499 | (GA)20 | F: AGAAGCACGAAAGAGCGTGT | 60-55 | 320 | Monomorphic |
|  |  |  | R: CGGTATTCCATTCGAACATAGG |  |  |  |
| IPAHM 308 | ER974500 | (GA)4GG(GA)31 | F: TTCATCGTTTCTGCGTGAAG |  | - | No amplification |
|  |  |  | R: TCAGTCGTCATTGTGCCTCT |  |  |  |
| IPAHM 309 | ER974501 | (GA)7GG(GA)34 | F: CTGGGAGGAAGAGAGAGCTG |  | - | No amplification |
|  |  |  | R: ACCAGCGGTGATGATAGAAA |  |  |  |
| IPAHM 317 | ER974502 | (AG)25 | F: GGGGATCTTGAGGACTTAACG |  | - | No amplification |
|  |  |  | R: AGCGGTGATGATAGAAACCAC |  |  |  |
| IPAHM 320 | ER974503 | (GA)11 | F: ACTTCGCGGGTGATAGAGTG | 65-60; 4 | 110 | Monomorphic |
|  |  |  | R: CGTCCCAATATCCCTTCAGA |  |  |  |
| IPAHM 323 | ER975404 | (CA)52 | F: CTCCACATCCTACCACAATCAA |  | - | No amplification |
|  |  |  | R: TCATCACCGGGGATTATTTG |  |  |  |
| IPAHM 324 | ER974505 | (AC)11 | F: ACGGCCAGTGAGCTGAAATA | 65-60; 1 | 110 | Monomorphic |
|  |  |  | R: AGTGGGAATTATGGGTAGCTAGG |  |  |  |
| IPAHM 327 | ER974506 | (GA)16 | F: CTGGGAGGAAGAGAGAGCTG |  | - | No amplification |
|  |  |  | R: CAAAGCCGTCATTACAACTACA |  |  |  |
| IPAHM 330 | ER974507 | (GT)8GA(GA)3 | F: TTCCCGATTGAACCTGAAAC | 65-60; 2 | 210 | Monomorphic |
|  |  |  | R: CTGCCATGTAGGAAGGGGTA |  |  |  |
| IPAHM 333 | ER974508 | (TG)15 | F: TGAGGGAATGATAGAAGGTGA | 65-60; 5 | 200 | Monomorphic |
|  |  |  | R: CATGTGTCCATTGTTACACCA |  |  |  |
| IPAHM 334 | ER974509 | (TC)10 | F: TCGAACAGGCTGTGATTCTG | 65-60; 1 | 310 | Monomorphic |
|  |  |  | R: TGCTGGAATGGAACTTTGCT |  |  |  |
| IPAHM 335a | ER974510 | (TC)3(AC)7AT(AC)4 | F: TTTTGACGACGTTCAGCTTG | 65-60; 4 | 180 | Monomorphic |
|  |  |  | R: CATCGCACATTGATTAACATCAC |  |  |  |
| IPAHM 335b | ER974511 | (CTCTTT)2CTT(CTTTT)4 | F: CCAGGCAGTTTGATTGTGTG | 65-60; 2 | 210 | Monomorphic |
|  |  |  | R: GTTCTTCTTAGCGGCAGAGC |  |  |  |
| IPAHM 342 | ER974512 | C16T(TC)9 | F: GGCATAAGGCATACACTCAGAA | 60-55; 4 | 150 | Monomorphic |
|  |  |  | R: CTCATGCTTTCCTTGGAAAAAG |  |  |  |
| IPAHM 352 | ER974513 | (GA)9GG(GA)8 | F: GGCGGGACACTTATCAGAGA | 65-60; 5 | 198 | Polymorphic |
|  |  |  | R: TTCCGTGTGACAGAAATACCC |  |  |  |
| IPAHM 354 | ER974514 | (GA)16 | F: TCCGAATCAAAATTGGCACT | 65-60; 5 | 190 | Polymorphic |
|  |  |  | R: ACCTCTCCCTCTCAAGTTTTTGT |  |  |  |
| IPAHM 356 | ER974515 | (GA)21G(GA)2 | F: TTGGGATTGGATCCCTAAGA | 65-60; 4 | 100 | Polymorphic |
|  |  |  | R: CAACTACCCTTCTCTCCACCA |  |  |  |
| IPAHM 357 | ER974516 | (AG)11 | F: GCATGCAAAGGAGAAAGCTC | 65-60; 4 | 140 | Monomorphic |
|  |  |  | R: GAGTGGATAATGAGGTGAACAAAG |  |  |  |
| IPAHM 372 | ER974517 | (GA)9 | F: GCCTTTTGAAATTCCCTTCC | 65-60; 1 | 180 | Monomorphic |
|  |  |  | R: TATGCCTCTTCCCCTTCCTT |  |  |  |
| IPAHM 373 | EE974518 | (TTG)6CT(GTT)8 | F: CAAGATCTTTCGTACATTCATTCAC | 65-60; 4 | 195 | Polymorphic |
|  |  |  | R: CACGCTCTTAGCAATTTCTGG |  |  |  |
| IPAHM 37 | ER974519 | (CA)10(TA)7 | F: CGTATGCATTATAAGTGCTCGACAA | 65-60; 2 | 215 | Polymorphic |
|  |  |  | R: AATCCGATATCCGCTTCGAC |  |  |  |
| IPAHM 376 | ER974520 | (CT)18 | F: GACGGTAATCGTGCCCTAAA |  | - | No amplification |
|  |  |  | R: TGTGAATGGTGAAGGAAGAAGA |  |  |  |
| IPAHM 377 | ER974521 | (GA)9 | F: AAGATGGAAACGGGAGCTTT | 65-60; 1 | 200 | Monomorphic |
|  |  |  | R: TGCAATACTCCCTCGCTTCT |  |  |  |
| IPAHM 395 | ER974522 | (GA)14 | F: CAGAGTCAATGGCAGCGTAG | 65-60; 4 | 395 | Polymorphic |
|  |  |  | R: TCCTTCCCTCATCTAAAACCAA |  |  |  |
| IPAHM 401 | ER974523 | (GA)9 | F: CTGATATTGAGATTGGAGTCACTG | 60-55; 5 | 100 | Monomorphic |
|  |  |  | R: TTCTCAAACTACCTCTTCTCTCCA |  |  |  |
| IPAHM 406 | ER974524 | (GA)9AA(GA)8 | F: TGAAAGGGATTGGACCAAAA | 65-60; 2 | 350 | Polymorphic |
|  |  |  | R: TGTTGGACAGGATTTCACACA |  |  |  |
| IPAHM 407a | ER974525 | (TC)7TGTT(TC)9 | F: TTGGTTGTTCGTAGCTCTGC | 65-60; 3 | 200 | Polymorphic |
|  |  |  | R: AGCACGGCAAACACTAACACT |  |  |  |
| IPAHM 407c | ER974526 | (GA)17 | F: TAACCAACACAAGCCCACAA | 65-60; 3 | 150 | Monomorphic |
|  |  |  | R: TCCATCATCACCCTCATCAA |  |  |  |
| IPAHM 409 | ER974527 | (GA)3CAGAGG (GA)3 | F: GGAGGGTGGTGTGAGGTAAC | 65-60; 1 | 130 | Monomorphic |
|  |  |  | R: ATCCACCGAAATCACAGCTC |  |  |  |
| IPAHM 412 | ER974528 | (TG)4CA(GA)10 | F: AAGCAAGCAAGCAAGCAAG |  | - | No amplification |
|  |  |  | R: AACAACTCACCACCACACCA |  |  |  |
| IPAHM 413 | ER974529 | (GA)13 | F: GGGGTCCTTCCTCTCTATGC |  | - | No amplification |
|  |  |  | R: TTTTCCCTCCTTCTTCAGTT |  |  |  |
| IPAHM 414 | ER974530 | (CT)2(TC)8TT(TC)8 | F: GATCGTCGCTTCTTTGCTCT | 65-60; 1 | 120 | Monomorphic |
|  |  |  | R: CCTTCCTAGAACAGCGATGG |  |  |  |
| IPAHM 415 | ER974531 | (GA)12 | F: TGGTCCATAGCTGCCTTTTT | 65-60; 2 | 205 | Monomorphic |
|  |  |  | R: GAGTGGAGGGGAATCTTCAA |  |  |  |
| IPAHM 416 | ER974532 | (TC)11 | F: GCGTTGTTCACGTTATTGGA |  | - | No amplification |
|  |  |  | R: ACCAGGTGGGAGACTCATTG |  |  |  |
| IPAHM 423 | ER974533 | (GA)7G5(TG)16 | F: TTATGCTTCTCTGTTTCACTTTT |  | - | No amplification |
|  |  |  | R: GAATATCAAGCTCCTAACTCAGC |  |  |  |
| IPAHM 429 | ER974534 | (GT)17 | F: GTGTGCGTGAGTGGGTAATG | 65-60; 5 | 600 | Polymorphic |
|  |  |  | R: TGAAATGAGCCAATCAGCTATC |  |  |  |
| IPAHM 451 | ER974535 | (TG)11 | F: AAAGAGAGGGAGCCCGTAAG | 65-60; 1 | 350 | Polymorphic |
|  |  |  | R: CCTGCGGGTAGGGTATATATTG |  |  |  |
| IPAHM 455 | ER974536 | (TA)5(TG)16 | F: TGCAGAGACTTGTATTTTGAGG | 65-60; 2 | 150 | Polymorphic |
|  |  |  | R: AAGCCTTTGCGAATATAACC |  |  |  |
| IPAHM 456 | ER974537 | (GT)11(GA)15 | F: TGGGGAAAGATTTCAGTCAGTT | 55-45; 3 | 260 | Monomorphic |
|  |  |  | R: TCTGCCGTTTCTTGCTTTTT |  |  |  |
| IPAHM 461 | ER974538 | (TG)9CG(TG)15 | F: TGAACCAGGTATCTGAAAAGTTG |  | - | No amplification |
|  |  |  | R: TTAACAAACCCGTTAGCAACAA |  |  |  |
| IPAHM 463 | ER974539 | (CA)10 | F: AGGTTGTAATGAGGCCAAGG |  | - | No amplification |
|  |  |  | R: TGGAAATTATGGGTAGTTAGGTGTG |  |  |  |
| IPAHM 466 | ER974540 | (TG)3CG(TG)7(GA)8 | F: AGCCCTTCCTTAAATGGATGTT | 65-60; 1 | 150 | Monomorphic |
|  |  |  | R: GAGCAGATTCTGATTTCTGCAA |  |  |  |
| IPAHM 467 | ER974541 | (TG)14AG(TG)7 | F: GTGTGTAGTACCCCACAGAGCA | 65-60; 3 | 400 | Monomorphic |
|  |  |  | R: ACGTGAAATCACAGAAAAGCAT |  |  |  |
| IPAHM 468 | ER974542 | (GA)15 | F: GGCTTTTGAAGTTCCCTTCC | 65-60; 4 | 200 | Polymorphic |
|  |  |  | R: TATGCCTCTTCCCCTTCCTT |  |  |  |
| IPAHM 473 | ER974543 | (GT)16 | F: AACAAGTTCGAAGGTGCATGT |  | - | No amplification |
|  |  |  | R: CACAGCCCCAAGGATTAGAA |  |  |  |
| IPAHM 474 | ER974544 | (GA)20 | F: CAAAGGGGAGCACAAACATAA | 65-60; 5 | 200 | Monomorphic |
|  |  |  | R: TCTTCTCACATGTCCAAACCA |  |  |  |
| IPAHM 475 | ER974545 | (GT)7(GA)12 | F: GTGATTTCCTGGTTGGTGCT | 65-60; 2 | 300 | Polymorphic |
|  |  |  | R: AGCCTCAGCTGGTTTTGCT |  |  |  |
| IPAHM 508 | ER974546 | (TG)14 | F: ATCACCTGGAAAATGTTCTGCT |  | - | No amplification |
|  |  |  | R: ATACCAAGATCCAAGCGAAGAA |  |  |  |
| IPAHM 509 | ER974547 | (CA)25 | F: GCCTGCAAAGCTAAGGTTG |  | - | No amplification |
|  |  |  | R: TGGAGAGTTAGGATTTCTGGCTA |  |  |  |
| IPAHM 524 | ER974548 | (GA)20AA(GA)3 | F: GCCATGGATAAGAACCTGAAA | 65-60; 1 | 300 | Polymorphic |
|  |  |  | R: CAGTAAGCTGAGCTGGCAGA |  |  |  |
| IPAHM 526 | ER974549 | (GA)16 | F: TGAGTCGCGGAGAGAAAAGT | 65-60; 2 | 205 | Monomorphic |
|  |  |  | R: TCCATCATTCTCACACATCACA |  |  |  |
| IPAHM 530 | ER974550 | (GA)10­AA(GA)16 | F: CAACATCTCACTTATCCAATTCGTC | 65-60; 1 | 215 | Monomorphic |
|  |  |  | R: TGATGCAATTCACCTTTCCTC |  |  |  |
| IPAHM 531 | ER974551 | (TAC)7 | F: TGCCAGGTTGCTGTAACAAA | 65-60; 1 | 305 | Polymorphic |
|  |  |  | R: CATACACGCTTTTCCCCTGT |  |  |  |
| IPAHM 532 | ER974552 | (CA)21 | F: CAAATTGCCCTTCTCTCCAC |  | - | No amplification |
|  |  |  | R: CGATCATGAGGTGCTAAAAGG |  |  |  |
| IPAHM 534 | ER974553 | (TG)20 | F: TTAGCCACAAAACAGCTTGG |  | - | No amplification |
|  |  |  | R: CTCCACAATAGACCAATTCACA |  |  |  |
| IPAHM 536 | ER974554 | (CA)8AA(CA)31 | F: CAGCACCACAATCTCCACAT | 65-60; 1 | 210 | Monomorphic |
|  |  |  | R: AGAGAGTGTTGTGGGGTGTAGTT |  |  |  |
| IPAHM 537 | ER974555 | (CA)16 | F: GGCCAACTGTGTTTACTGGAA |  | - | No amplification |
|  |  |  | R: TGGCAATCATCTTTGAGTCG |  |  |  |
| IPAHM 538 | ER974556 | (CA)14 | F: TGCGAGGAGAGCTAATTAAACA |  | - | No amplification |
|  |  |  | R: TGTTTGGTTGTGAATGTTGG |  |  |  |
| IPAHM 540 | ER974557 | (TA)4(TG)12 | F: TGGAGAACTAGGATCTCTTTTGTG | 65-60; 1 | 128 | Polymorphic |
|  |  |  | R: CCTAACTCAGCCTGCGAAAC |  |  |  |
| IPAHM 543 | ER974558 | (CA)30 | F: GGCACACCTAGTTAATGCACAA |  | - | No amplification |
|  |  |  | R: GGGTGAGGGTAAGGGAAAAG |  |  |  |
| IPAHM 556 | ER974559 | (TG)16 | F: CCCCAAGTCACGAATGTTTT |  | - | No amplification |
|  |  |  | R: CCACAATCAACCATACCTCAA |  |  |  |
| IPAHM 561 | ER974560 | (TG)11 | F: TGGTGCCACTTTATGTATTGGT |  | - | No amplification |
|  |  |  | R: GTCCATAGCCCCACCTGATA |  |  |  |
| IPAHM 562 | ER974561 | (GT)8 | F: TTCTAGTGACGCAGTCATGC |  | - | No amplification |
|  |  |  | R: TCACACATCATTCGCAATC |  |  |  |
| IPAHM 569 | ER974562 | (TG)23T5 | F: ATTGGTGGATATCGGACTCG | 65-60; 2 | 200 | Polymorphic |
|  |  |  | R: CCTATCCTTCAAGGCTTCTCG |  |  |  |
| IPAHM 577 | ER974563 | (GA)22 | F: TGAGAGAGAGAGACCGCACA |  | - | No amplification |
|  |  |  | R: CACCTATGTCCACGATTACGC |  |  |  |
| IPAHM 57 | ER974564 | (CA)19 | F: CAAATTGCCCTTCTCTCCAC |  | - | No amplification |
|  |  |  | R: AAGGGAGGGGTTAGATTGGA |  |  |  |
| IPAHM 586 | ER974565 | (CA)34 | F: TTGCAACGACGAGTCTATCGTA |  | - | No amplification |
|  |  |  | R: TTGAAATTGTTGCGAGGTAATG |  |  |  |
| IPAHM 589 | ER974566 | (CA)21 | F: TCAGCCTGCGAAACTAAGGT | 65-60; 2 | 300 | Monomorphic |
|  |  |  | R: ATGTGGATGCGTGTTGTGTT |  |  |  |
| IPAHM 600 | ER974567 | (CA)14CG(CA)4 (TA)3 | F: TCAGCCTGCGAAACTAAGGT |  | - | No amplification |
|  |  |  | R: TGAGTGTGATGAGTTGTGTAGGTAA |  |  |  |
| IPAHM 602 | ER974568 | (CA)22CG(CA)4 | F: CAGCCCAAATTTCTCGTTTC |  | - | No amplification |
|  |  |  | R: TGTTGTGGTATGAGACTAGTGCAA |  |  |  |
| IPAHM 606 | ER974569 | (CA)15TA(CA)3 (TA)4 | F: CCTAACTCAGCCTGCGAAAC | 65-60; 1 | 100 | Monomorphic |
|  |  |  | R: CAGAGGTGTTTGGAGAACTAGGA |  |  |  |
| IPAHM 637 | ER974570 | (TG)24 | F: GGCGTAAAGCTTTGTGTGGT |  | - | No amplification |
|  |  |  | R: CCTAACTCAGCCTGCGAAAC |  |  |  |
| IPAHM 638 | ER974571 | (TG)9TA(TG)9 | F: TGCCCCTAGGATTGAGTTTG |  | - | No amplification |
|  |  |  | R: TCAGCCTGCGAAACTAAGGT |  |  |  |
| IPAHM 659 | ER974572 | (GA)18 | F: AAGTCACTGGCCAAAACTGC | 65-60; 1 | 130 | Polymorphic |
|  |  |  | R: CCCTCGATTTCGACTCAGAC |  |  |  |
| IPAHM 678 | ER974573 | (GA)3GG(GA)21 | F: CCATCATCGTCATTGTGGAG |  | - | No amplification |
|  |  |  | R: GAGTGAGCTAGAGGGAAGCTCT |  |  |  |
| IPAHM 684 | ER974574 | TGTA(TG)10 | F: GCCCGAGTTTTGAAGACCTA | 65-60; 1 | 200 | Polymorphic |
|  |  |  | R: CAAGGCCTCAACTTCCCTAA |  |  |  |
| IPAHM 689 | ER974575 | (GA)20 | F: GATGACAATAGCGACGAGCA | 65-60; 2 | 240 | Polymorphic |
|  |  |  | R: GTAAGCCTGCAGCAACAACA |  |  |  |
| IPAHM 693 | ER974576 | (CT)27TCTT(TC)7T (TC)2 | F: TTTGCCGAAAAGGTGGTATC |  | - | No amplification |
|  |  |  | R: ATACACACCCCACCCCAATA |  |  |  |
| IPAHM 695 | ER974577 | (CT)6CG(CT)15(TG)3 | F: CCCACATGTGTATGCGTTTT | 65-60; 1 | 300 | Monomorphic |
|  |  |  | R: TGCATGCTAAGCCAAGCTAA |  |  |  |
| IPAHM 696 | ER974578 | (GA)3A(GA)26 | F: CTGGGAGGAAGAGAGAGCTG | 65-60; 2 | 180 | Monomorphic |
|  |  |  | R: CCATCATCACCCTCATCAAA |  |  |  |
| IPAHM 701 | ER974579 | (GA)7GG(GA)15(GT)11 | F: CTGGGAGGAAGAGAGAGCTG |  | - | No amplification |
|  |  |  | R: CATTGGGCATCAACCATACA |  |  |  |
| IPAHM 710 | ER974580 | (GA)21 | F: TGGCTCCCTAGCACTACTCAA |  | - | No amplification |
|  |  |  | R: CCCCTCCCCTTTCTTTCTCT |  |  |  |
| IPAHM 714 | ER974581 | (GA)21 | F: TGCATGGGAGGAAGAGAGAG |  | - | No amplification |
|  |  |  | GGGTATTGGACAAGGAAGGAA |  |  |  |
| IPAHM 716 | ER974582 | (GA)8 | F: CACTATGCCACGAGCTTCAA | 65-60; 1 | 210 | Polymorphic |
|  |  |  | R: ACACACCACAACCACAGAGC |  |  |  |
| IPAHM 718 | ER974583 | (GA)15 | F: AGATAGGGGCCGAGCTAGAG | 65-60; 2 | 230 | Polymorphic |
|  |  |  | R: ATGCTCCACCCCTTCATTTT |  |  |  |

*Reaction components: 1= 0.2µl of 10pM primer; 1µl of DNA (5ng/µl); 1µl of Mg^++^ (10mM); 0.1 µl of dNTP 2mM ; 0.2 µl of *Taq* DNA polymerase (0.5U/µl);

2= 0.2µl of 10pM primer; 2µl of DNA (5ng/µl); 2µl of Mg^++^ (10mM); 0.2µl of dNTP 2mM; 0.2µl of *Taq* DNA polymerase (0.5U/ µl)

3= 0.3µl of 10pM primer; 1µl of DNA (5ng/µl); 1.5µl of Mg^++^ (10mM); 0.2µl of dNTP 2mM; 0.5µl of *Taq* DNA polymerase (0.5U/µl)

4= 0.3µl of 10pM primer; 2µl of DNA (5ng/µl); 2µl of Mg^++^ (10mM); 0.1µl of dNTP 2mM; 0.3µl of *Taq* DNA polymerase (0.5U/µl)

5= 0.5µl of 10pM primer; 2µl of DNA (5ng/µl); 1µl of Mg^++^ (10mM); 0.2µl of dNTP 2mM; 0.5µl of *Taq* DNA polymerase (0.5U/µl)
